# Measurement Invariance of the Bergen Social Media Addiction Scale Across Genders

**DOI:** 10.3389/fpsyg.2022.879259

**Published:** 2022-06-21

**Authors:** Heng Yue, Xuemin Zhang, Xiangjuan Cheng, Bo Liu, Hugejiletu Bao

**Affiliations:** ^1^School of Psychology, Inner Mongolia Normal University, Hohhot, China; ^2^College of Physical Education, Inner Mongolia Normal University, Hohhot, China

**Keywords:** measurement invariance, Bergen Social Media Addiction Scale, social media addiction, gender difference, college students

## Abstract

Social media addiction has been a hot issue in scientific research in recent years, its antecedents and consequences have been extensively studied. Among these studies, Bergen Social Media Addiction Scale (BSMAS) is one of the most commonly used instruments. However, little is known about whether this scale has the equivalent psychometric properties for men and women. The purpose of the current study is to examine the measurement invariance (including configural invariance, metric invariance, scalar invariance, and error variance invariance) of the BSMAS across genders. In total, 1,120 participants were recruited from 5 universities. R program was applied to conduct the single-group and multiple-group confirmatory factor analysis (CFA) based on the social media addiction symptom ratings. The results demonstrated that BSMAS was a valid and psychometrically robust instrument for assessing the risk of social media addiction among university students, and that the four types of measurement invariance of the BSMAS across genders were confirmed. Consequently, gender differences in the BSMAS scores are likely to reflect the genuine differences between men and women, and comparisons on the level of social media addiction of university students between gender groups can be interpreted meaningfully.

## Introduction

Social media has brought great convenience to our daily life. It integrates many interesting and useful functions such as instant messaging, games, music, short video, and so on. These services not only allow individuals to connect with others irrespective of the constraints of time and space; but also provide numerous leisure activities, and make life rich and colorful. Therefore, many people tend to spend lots of time on social media, under this circumstance, they may be very likely to develop the problematic behavior–social media addiction. Social media addiction refers to the behavior characterized by being excessively concerned about social media activities and spending too much time and effort on these activities, at last, the work, study, interpersonal relationship relationships, mental health, and other important life areas were impaired (Schou Andreassen and Pallesen, 2014). At present, social media addiction has attracted the attention of both social scientists and the general public. In 2021, a meta-analysis across 32 countries revealed that the average prevalence rate of social media addiction had reached 24% (Cheng, 2021). The detrimental consequences of this addictive behavior have been confirmed as well. Previous studies indicated that social media addiction was positively associated with anxiety, depression, and other forms of psychological distress (Keles, 2020). Researchers have found that problematic social media use is related to sleep disturbances (Wong et al., 2020). Moreover, evidence also suggests that overreliance on social media brings about the impoverishment of social skills, short-term attention, and the decrease of the ability to keep information (Kuss and Griffiths, 2017). Therefore, it is of significant importance and has been an urgent problem to conduct in-depth research and seek feasible coping strategies on social media addiction.

In order to assess individuals' levels of social media addiction, some measurement instruments have been developed by scholars, such as Risk of Addiction to Social Networks Scale (Vilca and Vallejos, 2015), Social Media Disorder Scale (van den Eijnden et al., 2016), Addictive Tendencies Scale (Wilson et al., 2010), and Bergen Social Media Addiction Scale (BSMAS) (Andreassen et al., 2016). Among these instruments, BSMAS has been the most widely used. Up to now, this scale has been translated and applied in different languages, for instance, English (Andreassen et al., 2016), Italian (Monacis et al., 2017), Persian (Lin et al., 2017), and Chinese (Leung et al., 2020; Luo et al., 2021), the reliability and validity has been supported in these cultural contexts as well. BSMAS was adapted from the Bergen Facebook Addiction Scale (BFAS) (Andreassen et al., 2012), and the latter was developed based on the six core components (including salience, mood modification, tolerance, withdrawal, conflict, and relapse) of addictive behavior (Griffiths, 2005), and each item of BSMAS reflected one of these components. The higher the sum score of BSMAS, the greater the severity of being a social media addict.

By using BSMAS, researchers surveyed teenagers and draw the conclusion that the level of addiction in women were higher than men (Andreassen et al., 2016; Bányai et al., 2017; Chae et al., 2018; Su et al., 2020). The reason may lie in the fact that social interaction and cooperation are the main activities of their social media use (Andreassen et al., 2016), compared with men, women tend to seek feedback from social media, spend more time managing their online image, and soliciting assistance from their friends (Yau and Reich, 2019; Twenge and Martin, 2020), besides, women are more prone to acquire social media addiction when they experience empty and depression (Chae et al., 2018; Su et al., 2020). In this way, they are more likely to excessively use social media and ultimately develop social media addiction. Some scholars also applied this instrument to conduct their study in adolescent samples, the results demonstrated that males were more likely to be addicted in social media (Altin and Kivrak, 2018; Azizi et al., 2019; Shibli and Akhtar, 2020; Luo et al., 2021; Gao and Eissenstat, 2022). In these scholars' opinion, men may experience more negative effects and be more likely to have a poor academic performance, which will lead men to use social media for longer time as opposed to their female counterparts and contribute to their vulnerability of social media addiction (Shen, 2019; Bhandarkar et al., 2021; Gao and Eissenstat, 2022). Moreover, some studies also contended that there was no significant association between gender and the severity of social media addiction (Kirik et al., 2015; Ahmed et al., 2021; Cheng, 2021; Mahmood et al., 2022). This indicates that on average, men and women may have the identical level of social media addiction or exhibit the similar addictive symptoms, regardless of the motivations and justifications of their usage behavior.

However, these conclusions may be doubtable, because the perquisite of the comparison between men and women is that the measurement of the construct is equivalent in both the genders. In previous studies, due to the fact that measurement invariance tests of the BSMAS had not been conducted, the differences between men and women may come from the measurement bias, for example, the meaning of the BSMAS items may be different between genders. Therefore, researchers cannot draw the conclusion that gender disparities in social media addiction represent the real differences between men and women before establishing the measurement invariance, as they may be the result of systematic biases that men and women reply to the BSMAS items. From what has been mentioned earlier, it is necessary to examine the cross-gender measurement invariance of the BSMAS.

Measurement invariance refers the degree to which the contents of the scale are being perceived and interpreted in the identical way across different samples (Byrne and Watkins, 2003). Other scholars also indicated that measurement invariance could be considered as the extent to which measurements performed under different circumstances exhibit the same psychometric properties (Meade et al., 2008). Although there are some distinctions between the definitions, measurement invariance is regarded as the premise for comparing the differences across genders, ages, ethnicities, cultures, and so on (Chen, 2007). Because inference problems may occur and conclusions resulted from a study may be inaccurate or invalid if the measurement instruments we depend on do not have the identical meanings across different groups (Chen, 2007). In practice, measurement invariance is conducted *via* an iterative process, a series of hierarchically nested models is examined across meaningful participant groups, or across time, to determine to what extent the proposed factor structure fits across these populations (Collison et al., 2021). The procedures for assessing the measurement equivalence in the CFA framework has been classified into two parts: measurement invariance and structural invariance (Byrne et al., 1989; Vandenberg and Lance, 2000; Mengcheng, 2014). Measurement invariance tests the relationships between the observed variables and the latent constructs, this part includes four processes: configural invariance, metric invariance, scalar invariance, and error variance invariance; structural invariance examines the latent variables themselves; this part includes three processes: factor variance invariance, factor covariance invariance, and latent mean invariance (Byrne et al., 1989; Vandenberg and Lance, 2000; Mengcheng, 2014). Due to the fact that structural invariance represents very strict criterion that are often hardly to be satisfied in practice, therefore, measurement invariance is the most frequently examined part of equivalence (Chen, 2007). According to this, in the present study, we will test the four processes of measurement invariance in the statistical analysis part as well.

Previous studies examined the cross-culture and cross-time invariance of the BSMAS in university students (Chen et al., 2020a; Leung et al., 2020), results of these studies confirmed the measurement invariance of this inventory, in terms of cross-gender invariance of this scale, one study had verified the measurement equivalence of BSMAS between genders in primary school students (Chen et al., 2020b). Nevertheless, because there was a high-prevalence rate of social media addiction among college students (Tang and Koh, 2017), they had been the focus group of social media addiction (Ya-li et al., 2021), moreover, participants enrolled in most studies were college students (Cheng, 2021). Therefore, the aim of the present study is to test the cross-gender measurement invariance of BSMAS in college student populations.

## Method

### Participants

Participants were recruited from five universities in Inner Mongolia China. In total, 1,500 questionnaires were sent out during the participants' spare time, and 1,260 were returned. In total, 140 invalid ones were removed because these participants failed to complete their questionnaires or they did not provide their age or genders. Finally, data from 1,120 participants were used for the final analysis. There were 513 men and 607 women, the average age of the participants was 20.89 ± 1.40 years, ranging from 18 to 26 years of age.

### Measurement

Bergen Social Media Addiction Scale (BSMAS) was administered to assess the level of one's social media addiction. Participants were asked to report their experiences in the use of social media within a 12-month period. The BSMAS consisted of 6 items (e.g., “How often during the last year have you tried to cut down on the use of social media without success?”), each item reflected one of the core addiction components (salience, mood, modification, tolerance, withdrawal conflict, and relapse) proposed by Griffiths (Griffiths, 2005). A 5-point Likert scale was adopted for rating (from 1 = “very rarely” to 5 = “very often”), a higher sum score obtained from the BSMAS indicated a higher likelihood of being addicted to social media. In the present study, the Cronbach's alpha coefficient for the scale in the overall sample was 0.822, in male sample was 0.828; and in female sample was 0.817.

### Procedure

The current study was performed in accordance with the Declaration of Helsinki and was approved by the Ethics Committee of the College of Psychology Inner Mongolia Normal University. All the participants were randomly selected from five universities in Inner Mongolia; they should be older than or equal to 18 years and they should have at least one social media account (such as WeChat, QQ, Weibo, and other social media platform). Before sending out the questionnaires, all the participants were informed that the data were only used to conduct scientific research, they were not compelled to participate in the study; their responses were anonymous; they could drop out at any moment they would like and this would not impact their life and their performance in the final examination. Finally, after acquiring the informed consent of the participants and their teachers or supervisors, questionnaires were merely distributed to the students who were willing to participate in this study.

### Statistical Analysis

Microsoft Excel 2016 was employed to input the data, SPSS 25.0 was applied to conduct descriptive statistics, test the normality of the data, and calculate the Cronbach's alpha coefficient for the overall sample and each gender group. RStudio with the packages “lavaan” (Rosseel, 2012) and “semTools” (Jorgensen et al., 2018) was applied to perform the confirmatory factor analysis (CFA) and assess the measurement invariance. The normality of the data was evaluated by the values of skewness and kurtosis, the absolute values of the two indices smaller than 2 indicated that the scores of the items are normally distributed (Pituch and Stevens, 2015). Due to the ordinal nature of the BSMAS, according to the suggestions of the previous scholars, weighted least squares with mean and variance adjusted (WLSMV) estimator was employed to assess the model fit indexes (Wu and Estabrook, 2016). The goodness of model fit of CFA was estimated by multiple indices: χ^2^ statistic, Root Mean Square Error of Approximation (RMSEA), Comparative Fit Index (CFI) and Standardized Root Mean Square Residual (SRMR). For RMSEA, a value smaller than 0.05 represented a close fit; a value ranging from 0.07 to 0.08 indicated a fair fit; a value in the range of 0.08 to 0.10 denoted a mediocre fit (Browne and Cudeck, 1992; MacCallum RC, 1996). For CFI, the value above 0.90 was considered as acceptable (Hu and Bentler, 1999). For SRMR, the value smaller than 0.05 signified acceptable fit (Hu and Bentler, 1999; Shi and Maydeu-Olivares, 2020). Multiple-group CFA was conducted to test the measurement invariance of the social media addiction scale across groups of men and women. Four increasingly constrained multiple-group CFA processes were performed in an iterative manner. First, the configural invariance model was established as the baseline model to investigate whether the same factor structure was equivalent across groups. Second, the metric (or “weak”) invariance model was established to test whether the factor loadings of the items were the same for both men and women. Metric invariance is an important prerequisite for the meaningful comparison between gender groups (Bollen, 1989). Third, the scalar (or “strong”) invariance model was established to estimate whether the factor loadings and the intercepts were equivalent across men and women. Forth, the strict invariance model was established to test whether the items have the same factor loadings, intercepts, and residual variances in both gender groups. Because the four models mentioned earlier were nested hierarchically within the previous ones, measurement invariance was assessed by the overall model fit for each model and the changes in fit indices between the nested models. Due to the ordinal nature of the BSMAS, according to the recommendations of previous studies, measurement invariance was supported if the comparison between the two models fulfilled these criteria: a non-significant Δχ^2^, ΔRMSEA < 0.050, ΔCFI < 0.004, and ΔSRMR ≤ 0.01 (Rutkowski and Svetina, 2017; Clark, 2020).

## Results

### Descriptive Statistics

Results of descriptive statistics for the total sample and both gender groups were presented in Table 1. In the overall sample, the ranges of means and SDs for the BSMAS item scores ranged from 2.52 to 3.00 and 1.012 to 1.093, separately. In the male group, the means and SDs ranged from 2.50 to 2.96 and 0.994 to 1.132, separately; in the female group, the means and SDs ranged from 2.54 to 2.99 and 1.004 to 1.086, separately. All the absolute values of skewness and kurtosis were smaller than 2, this suggested the scores of the items approximately normally distributed (Pituch and Stevens, 2015). Because the maximum likelihood estimation was robust when the data followed an approximately normal distribution (Finney and DiStefano, 2006; Kaplan, 2008), therefore, the maximum likelihood estimation would be employed in the (multiple-group) CFA.

**Table 1:** Descriptive statistics of the BSMAS.

**Sample**	**Item**	**Mean**	**SD**	**Skewness**	**Kurtosis**
Total (*n =* 1120)	1	2.95	1.093	−0.013	−0.583
	2	2.69	1.082	0.210	−0.532
	3	2.93	1.072	0.135	−0.541
	4	3.00	1.028	−0.036	−0.465
	5	2.88	1.012	0.011	−0.361
	6	2.52	1.079	0.531	−0.303
Male (*n =* 513)	1	2.91	1.132	0.023	−0.692
	2	2.65	1.085	0.184	−0.566
	3	2.92	1.089	0.222	−0.577
	4	2.96	1.055	−0.021	−0.533
	5	2.86	0.994	−0.015	−0.330
	6	2.50	1.072	0.457	−0.417
Female (*n =* 607)	1	2.99	1.058	−0.036	−0.474
	2	2.72	1.080	0.235	−0.505
	3	2.93	1.057	0.056	−0.501
	4	3.04	1.004	−0.040	−0.402
	5	2.90	1.027	0.028	−0.385
	6	2.54	1.086	0.591	−0.216

### Single-Group Confirmatory Factor Analysis

Single-group CFA was conducted for the overall sample and individually for each gender. The unidimensional model was tested in the overall sample, male and female groups separately. Results of single-group CFA in the overall sample and each subsample were presented in Table 2. In the overall sample, the results indicated that the model fitted the data adequately, the fit indices were as follows: χ^2^ = 39.016, RMSEA = 0.055 (90% CI, 0.038–0.073), CFI = 0.994, SRMR = 0.018, the standardized factor loadings ranged from 0.637 to 0.753. In the male sample, fit indices of CFA suggested the model was appropriate: χ^2^ = 10.837, RMSEA = 0.020 (90%[CI], 0.000–0.056), CFI = 0.999, SRMR = 0.013, the standardized factor loadings ranged from 0.653 to 0.770. In the female sample, the outcomes also showed an acceptable model fit: χ^2^ = 51.820, RMSEA = 0.089 (90%[CI], 0.066–0.113), CFI = 0.983, SRMR = 0.030, the standardized factor loadings ranged from 0.629 to 0.735. In summary, results of CFA indicated the unidimensional model of BSMAS was acceptable for both gender groups.

**Table 2:** Results of single-group confirmatory factor analysis.

	**Robust Model Fit Indices**						**Standardized Item Factor Loadings**					
**Sample**	**χ^2^**	**df**	**RMSEA**	**RMSEA(90%CI)**	**CFI**	**SRMR**	**Item1**	**Item2**	**Item3**	**Item4**	**Item5**	**Item6**
Total (*n =* 1120)	39.016	9	0.055	0.038–0.073	0.994	0.018	0.656	0.736	0.637	0.753	0.693	0.698
Male (*n =* 513)	10.837	9	0.020	0.000–0.056	0.999	0.013	0.667	0.753	0.653	0.770	0.697	0.694
Female (*n =* 607)	51.820	9	0.089	0.066–0.113	0.983	0.030	0.648	0.722	0.629	0.735	0.691	0.702

### Measurement Invariance Across Genders

Multiple-group CFA was performed to examine the measurement invariance across genders. Fit indices for the four models and the differences between the pairs of nested models were displayed in Table 3.

**Table 3:** Fit indices for measurement invariance tests.

	**Robust Model Fit Indices**						**Model Difference**					
**Model**	**χ^2^**	**df**	**RMSEA**	**RMSEA (90%CI)**	**CFI**	**SRMR**	**ΔM**	**Δχ^2^**	**Δdf**	**ΔRMSEA**	**ΔCFI**	**ΔSRMR**
M1	64.947	18	0.068	0.051–0.087	0.991	0.023	–	–	–	–	–	–
M2	70.005	35	0.042	0.028–0.057	0.993	0.022	M2 VS. M1	5.058	17	−0.026	0.002	−0.001
M3	71.020	40	0.037	0.023–0.051	0.994	0.023	M3 VS. M2	1.015	5	−0.005	0.001	0.001
M4	71.681	46	0.032	0.016–0.045	0.995	0.023	M4 VS. M3	0.661	6	−0.005	0.001	0.000

*M1, configural invariance; M2, metric invariance; M3, scalar invariance; M4, strict invariance. All the Δχ^2^ was not significant*.

First, configural invariance was conducted by estimating the two gender groups with no equality constraint. The results indicated the configural invariance model (M1) fit the data well (χ^2^ = 64.947, RMSEA = 0.068 (90%[CI], 0.051–0.087), CFI = 0.991, SRMR = 0.023). Therefore, the configural invariance of the BSMAS was confirmed.

Second, metric invariance was performed through constraining the factor loadings to be the same between male and female groups. The fit indices suggested an acceptable model fit. Compared with M1, the metric invariance model (M2), Δχ^2^ was not significant, value changes of RMSEA (ΔRMSEA), CFI (ΔCFI), and SRMR (ΔSRMR) were −0.026 < 0.050, 0.002 < 0.004, −0.001 < 0.01, respectively, this indicated that the metric invariance of the BSMAS held across genders.

Third, scalar invariance was evaluated by restricting the factor loadings and intercepts of the items to be equal across the two gender groups. The results of the scalar invariance model (M3) demonstrated that the model fitted the data well (χ^2^ = 71.020, RMSEA = 0.037 (90%[CI], 0.023–0.051), CFI = 0.994, SRMR = 0.023). Compared with M2, the values of ΔRMSEA, ΔCFI, and ΔSRMR were all smaller than the recommended cutoff values for rejecting measurement invariance. The satisfaction of scalar invariance implied that the factor loadings and item intercepts were invariant for men and women.

Forth, strict invariance estimated through forcing the factor loadings, intercepts, and residual variances of the items to be the same across genders. The strict invariance model (M4) provided satisfactory fit indices (χ^2^/df = 71.681, RMSEA = 0.032 (90%[CI], 0.016–0.045), CFI = 0.995, SRMR = 0.023). Compared with M3, the values of ΔRMSEA, ΔCFI, and ΔSRMR were all within the recommended guidelines for supporting the strict invariance. Therefore, the strict invariance of the BSMAS across genders was confirmed.

## Discussion

Bergen Social Media Addiction Scale has been one of the most frequently used instruments for estimating the level of individuals' social media addiction. However, the cross-gender measurement invariance of this scale still needed to be investigated by the researchers. The present study examined the equivalence of psychometric properties of the BSMAS between men and women. The main findings and implications are presented as follows.

Outcomes from single-group CFA for the total sample, male and female sample all suggested that the unidimensional model of BSMAS provided a relatively good fit to the data. Similar to what has been reported in previous two studies (Leung et al., 2020; Huang et al., 2021), evidences yielded by the current research still indicated that the unidimensional factor structure of this scale was robust across genders. However, participants in one previous study were restricted to be university students from Hong Kong and Taiwan, understanding and writing Chinese traditional characters was one of the inclusion criteria (Leung et al., 2020). Although all of them are Chinese, there might still be some cultural differences between Hong Kong, Taiwan, and mainland China. Therefore, it was necessary to test the factor structure of BSMAS with participants who resided in mainland China. In fact, in the other previous study, researchers enrolled adolescents in mainland China to assess the factor structure of BSMAS. Nevertheless, when they performed CFA, they added the residual covariance path of item 1 and item 2 (Huang et al., 2021). From the viewpoint of statistics, this operation was not appropriate, because there was no theoretically defensible reason, and allowing correlated residuals might mask the latent pattern of the data (Landis et al., 2009). Therefore, the results of the present study still have significant importance.

In terms of the measurement invariance, results of single-group CFA and configural invariance analysis suggested that the BSMAS assessed the equivalent construct across genders. Findings from the multiple-group CFA demonstrated that the metric invariance of BSMAS between men and women was also held, this indicated that one unit change of the latent variable would be translated into the equivalent change of the particular observed variable in both the male and female groups (Widaman and Grimm, 2014). In practice, this meant that the differences of the severity of social media addiction between men and women could be interpreted directly, the same scores represented the identical meaning across genders (Fen, 2019). Scalar invariance of BSMAS was also supported across the two gender groups, this not only demonstrated that the item thresholds were invariant for men and women; but also indicated that a score of the latent variable would be converted into the same score on a particular observed variable in the two gender groups (Widaman and Grimm, 2014). Strict invariance of BSMAS was acceptable as well, this suggested that the residual variances of the manifest variables were homogeneous between the two groups. In other words, this meant that when predicting a given observed variable from the latent variable, the items of BSMAS had the identical error terms across the gender groups.

Results of the current study indicated that the unidimensional structure of the BSMAS was gender-invariant, and evidences of measurement invariance (including configural, metric, scalar, and strict invariance) of the BSMAS also suggested that the psychometric properties of this instrument were equivalent in both male and female groups. The same scores of the BSMAS reflected the identical severity of social media addiction across genders, and this might not be affected by the artifacts of measurement bias. Therefore, in Chinese college student populations, it is acceptable to use the BSMAS to compare the levels of social media addiction in different genders. This conclusion was similar to one previous study in which primary school students were enrolled as the participants (Chen et al., 2020b), although there are differences between the ages of the participants, the two studies all demonstrated the measurement invariance of the BSMAS across genders; and this further indicated that the discrepancies between men and women came from the differences of the latent construct, rather than the measurement bias of the BSMAS items. Besides, BSMAS was developed on the basis of the “components” model of addiction, therefore, results of the present study provide empirical evidence for this theory as well. On one hand, this study confirmed that social media addiction indeed shared these commonalities proposed by this theory, and these symptoms together with the structure of this model were shared and verified robust in mainland Chinese university students as well; on the other hand, these components were identical in both male and female genders, demonstrated that both groups had parallel interpretations on the item content in the BSMAS. The current study also had practical implications for researchers, clinicians, and the general public. They could compare the severity of social media addiction between the two gender groups directly without concerning about the measurement deviation.

## Conclusions

In the present study, the factor structure and the cross-gender measurement invariance of the BSMAS were examined, results indicated that the unidimensional structure and the measurement invariance of the BSMAS were supported. The differences of the BSMAS between gender groups are due to the differences of the latent construct, rather than the measurement bias of the items. Gender differences in the BSMAS are likely to reflect the genuine differences between men and women, and comparisons on the level of social media addiction of university students between gender groups can be interpreted meaningfully. Results of the current not only provides the empirical evidences for the structural validity of the BSMAS and for the “components” model of addiction in Chinese mainland culture, but also makes the researchers and the clinicians be more confident in the diagnosis, treatment and the related scientific research works of the addictive behavior between male and female university students.

## Data Availability Statement

The original contributions presented in the study are included in the article/supplementary material, further inquiries can be directed to the corresponding author/s.

## Ethics Statement

The studies involving human participants were reviewed and approved by College of Psychology Inner Mongolia Normal University. Written informed consent for participation was not required for this study in accordance with the national legislation and the institutional requirements.

## Author Contributions

HY, XZ, and HB: funding acquisition. XZ, XC, and BL: investigation. HY: writing—original draft. HB: writing—review and editing. All authors contributed to the article and approved the submitted version.

## Funding

This study is supported by four funds. (1) Inner Mongolia Normal University graduate students' research innovation fund (CXJJB20004). (2) Social Science Planning Project in Inner Mongolia, Evaluation System and Intervention Research of Adolescent Health Literacy in Inner Mongolia under Healthy China Strategy (2021NDB130). (3) Baotou Medical College's research project of Wen Xue, Wei Xue and Jian Xue, a study on emotional adjustment strategies and influencing factors of college students under the influence of public emergencies (2021BYWWJ-YB-37). (4) Inner Mongolia Natural Science Foundation, Exercise improves executive function in patients with chronic pain (2021MS03099).

## Conflict of Interest

The authors declare that the research was conducted in the absence of any commercial or financial relationships that could be construed as a potential conflict of interest.

## Publisher's Note

All claims expressed in this article are solely those of the authors and do not necessarily represent those of their affiliated organizations, or those of the publisher, the editors and the reviewers. Any product that may be evaluated in this article, or claim that may be made by its manufacturer, is not guaranteed or endorsed by the publisher.

## References

[B1] Ahmed O. Nayeem Siddiqua S. J. Alam N. Griffiths M. D. (2021). The mediating role of problematic social media use in the relationship between social avoidance/distress and self-esteem. Technol. Soc. 64, 101485. 10.1016/j.techsoc.2020.101485

[B2] Altin M. Kivrak A. O. (2018). The Social Media Addiction among Turkish University Students. J. Educ. Train. Stud. 6, 13–20. 10.11114/jets.v6i12.3452

[B3] Andreassen C. S. Billieux J. L. Griffiths M. D. Kuss D. J. Demetrovics Z. Mazzoni E. (2016). The relationship between addictive use of social media and video games and symptoms of psychiatric disorders: a large-scale cross-sectional study. Psychol. Addict. Behav. 30, 252–62. 10.1037/adb0000160 26999354

[B4] Andreassen C. S. Torsheim T. Brunborg G. S. Pallesen S. (2012). Development of a Facebook addiction scale. Psychol. Rep. 110, 501–17. 10.2466/02.09.18.PR0.110.2.501-517 22662404

[B5] Azizi S. M. Soroush A. Khatony A. (2019). The relationship between social networking addiction and academic performance in Iranian students of medical sciences: a cross-sectional study. BMC Psychol. 7, 28. 10.1186/s40359-019-0305-0 31053171 PMC6500070

[B6] Bányai F. Zsila Á. Király O. Maraz A. Elekes Z. Griffiths M. D. (2017). Problematic social media use: results from a large-scale nationally representative adolescent sample. PLoS ONE. 12, e0169839. 10.1371/journal.pone.0169839 28068404 PMC5222338

[B7] Bhandarkar A. M. Pandey A. K. Nayak R. Pujary K. Kumar A. (2021). Impact of social media on the academic performance of undergraduate medical students. Med. J. Armed Forces India. 77, S37–S41. 10.1016/j.mjafi.2020.10.021 33612930 PMC7873710

[B8] Bollen K. A. (1989). Structural Equations with Latent Variables. NJ: Wiley. 10.1002/9781118619179

[B9] Browne M. W. Cudeck R. (1992). Alternative ways of assessing model fit. Sociol. Methods Res. 21, 230–58. 10.1177/0049124192021002005

[B10] Byrne B. M. Shavelson R. J. Muthén B. (1989). Testing for the equivalence of factor covariance and mean structures: the issue of partial measurement invariance. Psychol. Bull. 105, 456. 10.1037/0033-2909.105.3.456

[B11] Byrne B. M. Watkins D. (2003). The issue of measurement invariance revisited. J. Cross-Cult. Psychol. 34, 155–75. 10.1177/0022022102250225

[B12] Chae D. Kim H. Kim Y. A. (2018). Sex differences in the factors influencing Korean college students' addictive tendency toward social networking sites. Int. J. Mental Health Addict. 16, 339–50. 10.1007/s11469-017-9778-3

[B13] Chen F. F. (2007). Sensitivity of goodness of fit indexes to lack of measurement invariance. Struct. Eq. Model. Multidiscip. J. 14, 464–504. 10.1080/10705510701301834

[B14] Chen I-. H. Ahorsu D. K. Pakpour A. H. Griffiths M. D. Lin C-. Y. Chen C-. Y. (2020b). Psychometric properties of three simplified Chinese online-related addictive behavior instruments among mainland Chinese primary school students. Front. Psychiatry. 875. 10.3389/fpsyt.2020.00875 33101070 PMC7495180

[B15] Chen I. H. Strong C. Lin Y-. C. Tsai M-. C. Leung H. Lin C-. Y. (2020a). Time invariance of three ultra-brief internet-related instruments: Smartphone Application-Based Addiction Scale (SABAS), Bergen Social Media Addiction Scale (BSMAS), and the nine-item Internet Gaming Disorder Scale- Short Form (IGDS-SF9) (Study Part B). Addict. Behav. 101, 105960 10.1016/j.addbeh.2019.04.018 31072648

[B16] Cheng C. Lau Y-c Chan L Luk JW. (2021). Prevalence of social media addiction across 32 nations: meta-analysis with subgroup analysis of classification schemes and cultural values. Addict. Behav. 117, 106845. 10.1016/j.addbeh.2021.106845 33550200

[B17] Clark J. C. (2020). Evaluating Model Fit for Longitudinal Measurement Invariance with Ordered Categorical Indicators: Brigham Young University.

[B18] Collison K. L. South S. Vize C. E. Miller J. D. Lynam D. R. (2021). Exploring gender differences in Machiavellianism using a measurement invariance approach. J. Pers. Assess. 103, 258–66. 10.1080/00223891.2020.1729773 32130029

[B19] Fen R Jun-liang L Yu-shang F Meng-cheng W. (2019). Measurement Invariance of the CES-D in Adult Sample. Chin. J. Clin. Psychol. 27, 973–7.+1061.

[B20] Finney S. J. DiStefano C. (2006). Non-normal and categorical data in structural equation modeling, in Structural Equation Modeling: A Second Course, eds Hancock G. R. Mueller R.O. (Greenwich, CT: Information Age Publishing), 269–314.

[B21] Gao N. Eissenstat S. J. DeMasi M. (2022). A one-year follow-up study of changes in social media addiction and career networking among college students with disabilities. J. Am. Coll. Health. 1–8. 10.1080/07448481.2022.2047707 35298365

[B22] Griffiths M. (2005). A ‘components’ model of addiction within a biopsychosocial framework. J. Substance Use. 10, 191–7. 10.1080/14659890500114359

[B23] Hu L. Bentler P. M. (1999). Cutoff criteria for fit indexes in covariance structure analysis: Conventional criteria versus new alternatives. Struct. Eq. Model. Multidiscip. J. 6, 1–55. 10.1080/10705519909540118

[B24] Huang L. Zhang J. Duan W. He L. (2021). Peer relationship increasing the risk of social media addiction among Chinese adolescents who have negative emotions. Curr. Psychol. 20, 1–9 10.1007/s12144-021-01997-w

[B25] Jorgensen T. D. Pornprasertmanit S. Schoemann A. M. Rosseel Y. Miller P. Quick C. (2018). semTools: Useful tools for structural equation modeling. R package version 05-1.

[B26] Kaplan D. (2008). Structural Equation Modeling: Foundations and Extensions. USA: Sage Publications.

[B27] Keles B. (2020). McCrae N, Grealish A. A systematic review: the influence of social media on depression, anxiety and psychological distress in adolescents. Int. J. Adolesc. Youth. 25, 79–93. 10.1080/02673843.2019.1590851

[B28] Kirik A. Arslan A. Çetinkaya A. Mehmet G. (2015). A quantitative research on the level of social media addiction among young people in Turkey. Int. J. Sport Cult. Sci. 3, 108–22. 10.14486/INTJSCS444

[B29] Kuss D. J. Griffiths M. D. (2017). Social networking sites and addiction: Ten lessons learned. Int. J. Environ.Res. Public Health. 14, 311. 10.3390/ijerph14030311 28304359 PMC5369147

[B30] Landis R. S. Edwards B. D. Cortina J. M. (2009). On the practice of allowing correlated residuals among indicators in structural equation models. In: Statistical and Methodological Myths and Urban Legends: Doctrine, Verity and Fable in the Organizational and Social Sciences. 2009. p. 193–214. New York: Routledge.

[B31] Leung H. Pakpour A. H. Strong C. Lin Y-. C. Tsai M-. C. Griffiths M. D. (2020). Measurement invariance across young adults from Hong Kong and Taiwan among three internet-related addiction scales: Bergen social media addiction scale (BSMAS), smartphone application-based addiction scale (SABAS), and internet gaming disorder scale-short form (IGDS-SF9) (study Part A). Addict. Behav. 101, 105969. 10.1016/j.addbeh.2019.04.027 31078344

[B32] Lin C-. Y. Broström A. Nilsen P. Griffiths M. D. Pakpour A. H. (2017). Psychometric validation of the Persian Bergen Social Media Addiction Scale using classic test theory and Rasch models. J. Behav. Addict. 6, 620–9. 10.1556/2006.6.2017.071 29130330 PMC6034942

[B33] Luo T. Qin L. Cheng L. Wang S. Zhu Z. Xu J. (2021). Determination the cut-off point for the Bergen social media addiction (BSMAS): diagnostic contribution of the six criteria of the components model of addiction for social media disorder. J. Behav. Addict. 10.1556/2006.2021.00025 34010148 PMC8996805

[B34] MacCallum RC Browne MW Sugawara HM. (1996). Power analysis and determination of sample size for covariance structure modeling. Psychol. Methods. 1, 130. 10.1037/1082-989X.1.2.130

[B35] Mahmood Q. K. Jafree S. R. Sohail M. M. (2022). Pakistani Youth and Social Media Addiction: the Validation of Bergen Facebook Addiction Scale (BFAS). Int.J. Mental Health Addict. 20, 581–94. 10.1007/s11469-020-00391-0

[B36] Meade A. W. Johnson E. C. Braddy P. W. (2008). Power and sensitivity of alternative fit indices in tests of measurement invariance. J. Appl. Psychol. 93, 568. 10.1037/0021-9010.93.3.568 18457487

[B37] Mengcheng W. (2014). Latent Variable Modeling Using. Mplus: Chong Qing University Press.

[B38] Monacis L. De Palo V. Griffiths V. Sinatra M. D. Social M. (2017). networking addiction, attachment style, and validation of the Italian version of the Bergen Social Media Addiction Scale. J. Behav. Addict. 6, 178–86. 10.1556/2006.6.2017.023 28494648 PMC5520120

[B39] Pituch K. A. Stevens J. P. (2015). Applied Multivariate Statistics for the Social Sciences: Analyses with SAS and IBM's. SPSS: Routledge. 10.4324/9781315814919

[B40] Rosseel Y. (2012). lavaan: An R package for structural equation modeling. J. Stat. Sofw. 48, 1–36. 10.18637/jss.v048.i0225601849

[B41] Rutkowski L. Svetina D. (2017). Measurement invariance in international surveys: Categorical indicators and fit measure performance. Appl. Measure. Educ. 30, 39–51. 10.1080/08957347.2016.1243540

[B42] Schou Andreassen C. Pallesen S. (2014). Social network site addiction-an overview. Curr. Pharmaceut. Des. 20, 4053–61. 10.2174/13816128113199990616 24001298

[B43] Shen J. (2019). Social-media use and academic performance among undergraduates in biology. Biochem. Mol. Biol. Educ. 47, 615–9. 10.1002/bmb.21293 31454138

[B44] Shi D. Maydeu-Olivares A. (2020). The effect of estimation methods on SEM fit indices. Educ. Psychol. Measure. 80, 421–45. 10.1177/0013164419885164 32425213 PMC7221491

[B45] Shibli N. Akhtar N. (2020). Affects of Social Media Addiction on Sleep Quality among Youth across. Cultures. 10.31124/advance.1231888733668732

[B46] Su W. Han X. Wu Y. u. H. Potenza Y. Do M. N. (2020). men become addicted to internet gaming and women to social media? A meta-analysis examining gender-related differences in specific internet addiction. Comput. Human Behav. 113, 106480. 10.1016/j.chb.2020.106480

[B47] Tang C. S.-K. Koh Y. Y. W. (2017). Online social networking addiction among college students in Singapore: comorbidity with behavioral addiction and affective disorder. Asian J. Psychiatry. 25, 175–8. 10.1016/j.ajp.2016.10.027 28262144

[B48] Twenge J. M. Martin G. N. (2020). Gender differences in associations between digital media use and psychological well-being: Evidence from three large datasets. J. Adolesc. 79, 91–102. 10.1016/j.adolescence.2019.12.018 31926450

[B49] van den Eijnden R. J. J. M. Lemmens J. S. Valkenburg P. M. (2016). The Social Media Disorder Scale. Comput. Human Behav. 61, 478–87. 10.1016/j.chb.2016.03.038

[B50] Vandenberg R. J. Lance C. E. A. (2000). review and synthesis of the measurement invariance literature: Suggestions, practices, and recommendations for organizational research. Organiz. Res. Meth. 3, 4–70. 10.1177/109442810031002

[B51] Vilca L. W. Vallejos M. (2015). Construction of the risk of addiction to social networks scale (Cr. ARS). Comput. Human Behav. 48, 190–8. 10.1016/j.chb.2015.01.049

[B52] Widaman K. F. Grimm K. J. (2014). Advanced Psychometrics Confirmatory Factor Analysis, Item Response Theory, and the Study of Measurement Invariance.

[B53] Wilson K. Fornasier S. White K. M. (2010). Psychological predictors of young adults' use of social networking sites. Cyberpsychol. Behav. Soc. Network. 13, 173–7. 10.1089/cyber.2009.0094 20528274

[B54] Wong H. Y. Mo H. Y. Potenza M. N. Chan M. N. M. Lau W. M. Chui T. K. (2020). Relationships between severity of internet gaming disorder, severity of problematic social media use, sleep quality and psychological distress. Int. J. Environ. Res. Public Health. 17, 1879. 10.3390/ijerph17061879 32183188 PMC7143464

[B55] Wu H. Estabrook R. (2016). Identification of confirmatory factor analysis models of different levels of invariance for ordered categorical outcomes. Psychometrika. 81, 1014–45. 10.1007/s11336-016-9506-0 27402166 PMC5458787

[B56] Ya-li Z. Yu-meng C. Juan-juan J. Guo-liang Y. (2021). The relationship between fear of missing out and social media addiction: a cross-lagged analysis. Chin. J. Clin. Psychol. 29, 1082–5. 10.16128/j.cnki.1005-3611.2021.05.039

[B57] Yau J. C. Reich S. M. (2019). “It's Just a Lot of Work”: Adolescents' Self-Presentation Norms and Practices on Facebook and Instagram. J. Res. Adolesc. 29, 196–209. 10.1111/jora.12376 29430759

